# The actions of *Pasteurella multocida* toxin on neuronal cells^☆^

**DOI:** 10.1016/j.neuropharm.2013.09.005

**Published:** 2014-02

**Authors:** Susan M. Surguy, Denise A. Duricki, Joanne M. Reilly, Alistair J. Lax, Jon Robbins

**Affiliations:** a Wolfson Centre for Age Related Diseases, King's College London, Guy's Campus, London SE1 1UL, UK; b Neuroscience, Physiology and Pharmacology, University College London, London WC1E 6BT, UK; c Department of Microbiology, Dental Institute, King's College London, Guy's Campus, London SE1 1UL, UK

**Keywords:** *Pasteurella multocida* toxin, G-protein, M-current, Kv7 channels, Calcium current, Intracellular calcium, Neurones, Superior cervical ganglion cell, NG108-15 cells, Muscarinic receptors, P2Y receptors

## Abstract

*Pasteurella multocida* toxin (PMT) activates the G-proteins Gα_i__(1-3)_, Gα_q_, Gα_11,_ Gα_12_ and Gα_13_ by deamidation of specific glutamine residues. A number of these alpha subunits have signalling roles in neurones. Hence we studied the action of this toxin on rat superior cervical ganglion (SCG) neurones and NG108-15 neuronal cells. Both Gα_q_ and Gα_11_ could be identified in SCGs with immunocytochemistry. PMT had no direct action on Kv7 or Cav2 channels in SCGs. However PMT treatment enhanced muscarinic receptor mediated inhibition of M-current (Kv7.2 + 7. 3) as measured by a 19-fold leftward shift in the oxotremorine-M concentration–inhibition curve. Agonists of other receptors, such as bradykinin or angiotensin, that inhibit M-current did not produce this effect. However the amount of PIP_2_ hydrolysis could be enhanced by PMT for all three agonists. In a transduction system in SCGs that is unlikely to be affected by PMT, Go mediated inhibition of calcium current, PMT was ineffective whereas the response was blocked by pertussis toxin as expected.

M1 muscarinic receptor evoked calcium mobilisation in transformed NG108-15 cells was enhanced by PMT. The calcium rises evoked by uridine triphosphate acting on endogenous P2Y_2_ receptors in NG108-15 cells were enhanced by PMT. The time and concentration dependence of the PMT effect was different for the resting calcium compared to the calcium rise produced by activation of P2Y_2_ receptors. PMT's action on these neuronal cells would suggest that if it got into the brain, symptoms of a hyperexcitable nature would be seen, such as seizures.

## Highlights

• *Pasteurella multocida* toxin (PMT) activates a range of G-protein alpha subunits.
• PMT increased muscarinic receptor mediated suppression of Kv7 potassium current in sympathetic neurones.
• PMT enhances both muscarinic and purinergic receptor mediated calcium mobilisation in NG108-15 cells.
• Both these events are mediated by the G-proteins Gq or G11.
• We would predict that the symptoms of central nervous system PMT toxicity would be hyperexcitable events such as seizures.

## 1 Introduction

*Pasteurella multocida* infections in humans are rare and if they occur it is usually due to contamination from a domestic animal. However there have been a number of reports of *P. multocida* meningitis (Green et al., 2002; O'Neill et al., 2005; Kawashima et al., 2010; Guet-Revillet et al., 2013) in which neurological complications are seen in between 17 and 43% of cases, most of which are seizures (Nakwan et al., 2009; Guet-Revillet et al., 2013). Of further concern is the possible link between the *P. multocida* infection and cancer (Lax, 2012).

The main virulent agent of *P. multocida* is a 146 kDa, 1285 amino acid, protein called *P. multocida* toxin (PMT). Not all *P. multocida* isolates are toxigenic, although very few human isolates of *P. multocida* have been tested for toxigenicity (Holst et al., 1992; Donnio et al., 1991). The toxigenic status of the isolates linked with neurological infections is not known. To date PMT actions have been investigated using a range of non-neuronal cells such as Swiss 3T3 (Staddon et al., 1991; Babb et al., 2012), HEK 293 (Repella et al., 2011), COS-7 (Waldron et al., 2012), CHO (Bünemann et al., 2000) and murine embryonic fibroblast (Orth et al., 2009) cell lines. However there is very limited knowledge on the effects of this toxin on neurones or neuronal cells, with the exception of the work of Brothers et al. (2011) on membrane interactions of PMT in mouse neuroblastoma × rat glioma hybrid (NG108-15) cells. Here we have investigated the cellular effect of PMT on primary neurones in culture (rat superior cervical sympathetic ganglion (SCG) cells) and a differentiated neuronal cell line (NG108-15).

PMT is a monomeric protein which has been sequenced, its functional domains analysed and a crystal structure obtained. The N-terminal region contains the binding and translocation domain that leads to its endocytosis (Baldwin et al., 2004; Pullinger et al., 2001). The C-terminal region contains the catalytic site and amino acid residues C1165, H1205 and H1223 are essential for its activity (Ward et al., 1998; Pullinger and Lax, 2007; Orth et al., 2003; Kitadokoro et al., 2007). The crystal structure suggests that the protein has three subdomains C1, C2 and C3. C1 (the most N-terminal region) has similarity with *Clostridium difficile* toxin B, and, consistent with the above, seems to be involved in membrane targeting. The other subdomains contain the catalytic and molecular recognition sites (Kitadokoro et al., 2007).

PMT is a mitogen for fibroblasts and activates a range of intracellular signalling pathways including PLC-β-mediated phosphoinositide turnover (Staddon et al., 1991; Wilson et al., 1997: Seo et al., 2000), Rho (Blocker et al., 2006) and mTORC1 (Oubrahim et al., 2013a, 2013b). Like pertussis and cholera toxins, PMT's molecular target are G-protein alpha subunits, in particular Gαq, Gα12, Gα13, Gαi (1–3) and Gα11 (Orth and Aktories, 2010; Orth et al., 2013). Unlike pertussis and cholera toxins the action of PMT on the G-protein alpha subunit is not ADP-ribosylation but deamidation of a glutamine residue (Orth et al., 2009, 2013; Babb et al., 2012). Here, in both SGC neurones and NG108-15 cells we have studied a number of signal transduction pathways to investigate the likely action(s) of the PMT on neurones.

## 2 Materials and methods

### 2.1 SCG cell culture

Sprague–Dawley rats (P17) were asphyxiated with rising CO_2_ and decapitated. The superior cervical sympathetic ganglia (SCG) were removed and placed in collagenase (500 U ml^−1^, Sigma, Poole, UK) for 15 min followed by trypsin (1  mg ml^−1^, Sigma) for 30 min. Ganglia were triturated with fire-polished glass pipettes, spun down, resuspended and plated onto either laminin (Sigma)-coated coverslips or 35 mm plastic dishes. Cultures were kept for up to 7 days at 37 °C (5% CO_2_) in L-15 medium supplemented with 10% foetal bovine serum, 2 mM glutamine, 24 mM NaHCO_3_, 38 mM glucose, 50 U ml^−1^ penicillin-streptomycin and 25 ng ml^−1^ nerve growth factor (Tocris, Avonmouth, UK). Unless otherwise indicated the materials were from Invitrogen (Paisley, UK).

### 2.2 NG108-15 cell culture

M1 muscarinic acetylcholine receptor transformed NG108-15 cells were cultured as described previously (Robbins et al., 1993; Bowden et al., 1999). Briefly cells were cultured in DMEM (supplemented with 5% FCS, HAT, penicillin & streptomycin), differentiated with PGE_1_ (10 μM) and IBMX (50 μM) and maintained in an incubator at 37 °C and 10% CO_2_.

### 2.3 Immunocytochemistry

SCG cells were plated onto coverslips and preincubated with 500 ng ml^−1^ PMT or vehicle for 24 h at 37 °C. The cells were washed in phosphate buffered saline (PBS, 3 × 5 min) and fixed in 4% paraformaldehyde in 0.1 M phosphate buffer for 30 min. The reaction was then quenched with 0.37% glycine and 0.27% ammonium chloride in PBS for 2 × 10 min, the cells were washed in PBS (3 × 5 min) and permeabilised with 0.1% Triton-X in PBS for 15 min. The blocking step was carried out with 2% bovine serum albumin (BSA) and 2% foetal calf serum in PBS for 60 min, following which the cells were washed in 1 mg ml^−1^ BSA in PBS (PBSBSA, 3 × 5 min) and exposed to the primary antibodies overnight at 4 °C. Four control and 4 PMT treated coverslips were exposed to an N-terminal antiGαq antibody (rabbit anti mouse/rat/human, Santa Cruz Biotechnology Inc, Santa Cruz, CA) at a dilution of 1:200 in PBSBSA. Similarly 4 control and 4 treated coverslips were exposed to an antiGα11 antibody (rabbit anti mouse/rat/human, Santa Cruz) at the same dilution. After washing in PBSBSA (6 × 5 min) cells were exposed to a secondary donkey anti rabbit antibody (Alexa 488 FITC, Invitrogen) at a dilution of 1:1000 in PBSBSA for 60 min at room temperature. Cells were then washed in PBSBSA (5 × 3 min), PBS (3 × 5 min) and deionised water (3 × 5 min). The coverslips were then mounted with fluorescent mounting medium (DAKO) on glass slides (VWR International, Poole, UK) and sealed with nail varnish (Rimmel, London, UK). Fluorescence was observed on the microscope/camera system detailed above with the excitation wavelength set at 480 nm, zero pixel binning and ×40 objective.

### 2.4 Electrophysiology

For potassium current recording, SCG cells that had been plated on 35 mm Petri dishes were superfused at room temperature (21–23 °C) with a solution containing (mM): NaCl (144), KCl (2.5), MgCl_2_ (0.5), CaCl_2_ (2), HEPES (5), glucose (10), tetrodotoxin (0.0005), pH 7.4 with Tris base, and 291 mOsM. Patch electrodes were fabricated from thin walled borosilicate glass (Harvard, Edenbridge, UK) on a 2 stage puller (PC-10; Narishigie, London, UK) polished to 1.5–3.0 MΩ (Narishigie NF-9). Electrodes were filled with a solution containing (mM): K acetate (80), KCl (30), HEPES (40), MgCl_2_ (3) EGTA (3), CalCl_2_ (1), pH 7.2 with NaOH (16), 270 mOsM. Cell membranes were permeabilised with freshly-made ampotericin B (1 mg 20 μl^−1^ DMSO, Sigma) and added to the pipette solution at 2  μl ml^−1^. After the series resistance had reduced to at least 15 MΩ (typically 6–12 MΩ) currents were recorded with an Axopatch 200A amplifier (Axon Instruments, Foster City, CA) and displayed on a PC running PClamp 8 via a preamplifier (Cyberamp 320, Axon Instruments) and an analogue-to-digital converter (Digidata 1200, Axon Instruments). Currents were filtered at 0.6 KHz and sampled at 10 KHz. Junction potentials measured at the end of the recordings were around −1 mV and were small enough not to be corrected for. For calcium channel current recordings the cells were plated onto dishes coated with poly-l-lysine to reduce the speed of neurite outgrowth and thereby improve the cells' spatial electrical characteristics. The CaCl_2_ in the superfusate was replaced by BaCl_2_ (10 mM) and the pipette solution consisted of (mM): CsCl (110), HEPES (40), MgCl_2_ (3), BAPTA (20), tetrodotoxin (0.0005), pH 7.3 with CsOH, 269 mOsM. BAPTA (20 mM) was used to suppress voltage-independent (non-G_o_-mediated) inhibition (Beech et al., 1991). Calcium channel currents were recorded in whole cell mode using a dual pulse protocol in which cells were clamped at −70 mV then depolarised twice to 0 mV for 100 ms each with an intervening strong depolarisation to +90 mV for 50 ms to evaluate voltage-dependence of the inhibition (Hille, 1994).

### 2.5 GFP-PLCδ-PH imaging

Intranuclear injections of SCG cells with the cDNA plasmid for the pleckstrin homology domain of phospholipase C-δ1 in an eGFP-C1 vector (GFP-PLCδ-PH, kindly provided by T. Meyer, Stanford University, USA; see Stauffer et al., 1998) were performed one day after culturing. The GFP-PLCδ-PH plasmid (100 μg μl^−1^) was dissolved in a solution containing (mM) NaCl (154) HEPES (5) KCl (2.5) MgCl_2_ (0.5), pH 7.4 (1 M, NaOH) and filtered (0.2 μm) by centrifugation at 13,000 rpm. Cells were impaled with 50 MΩ electrodes using an automated micro-injector (Transjector 5246, Eppendorf, Hamburg, Germany) and after injections were returned to the incubator for 24 h. Fluorescence recordings were performed on an inverted microscope as used for the calcium imaging. Cells were excited at 475 nm with a monochromator (TILL-optoelektronics); emission wavelength was 530 nm. The images were acquired by a 12-bit digital camera (C4880/80, Hamamatsu) and processed using a FITC pseudo-colour palette applied to the grey-scale images. Images were taken at around one per second with exposure times of 300–800 ms. Digital deconvolution (0.5 μm steps) was performed on-line with a nearest neighbour algorithm using Openlab software (Improvision).

### 2.6 Intracellular calcium measurement

SCG and NG108-15 cells on glass coverslips were incubated in the dark with Indo-1 AM (5 μM, 1 h, 37 °C), placed on a stage of a fluorescent microscope and superfused with a buffer solution containing (mM): NaCl 120.0, KCl 3.0, MgCl_2_ 1.2, NaHCO_3_ 22.6, glucose 11.1, HEPES 5.0, CaCl_2_ 2.5, CdCl_2_ 0.1, pH 7.4. UTP, UDP or oxotremorine-M was applied via the superfusate at room temperature. Bright cells were chosen under the microscope and UTP, UDP or oxotremorine-M was each applied for 5 s in order to generate a non-cumulative concentration response curves. Receptor agonists were applied in the presence of CdCl_2_ (100 μM) to prevent activation of voltage gated calcium channels. By using a photometric dual emission system, fluorescence was determined from single cells loaded with the dye Indo-1 (Grynkiewicz et al., 1985). Initially, an area of the coverslip with no cells present was used to offset background light levels and this was routinely checked and adjusted between cells and dishes. To carry out the measurements of Indo-1, the excitation light was passed through a neutral-density filter and a dichotic mirror, emitted light was passed through a 510 nm barrier filter. [Ca^2+^]_i_ was assessed by measuring the 405/488 nm emission ratio, which was then viewed on a personal computer running pClamp6 software. Ratiometric measurements (*R*) were converted to intracellular calcium concentrations ([Ca^2+^]_i_) using the equation: [Ca]_i_ = β*Kd*(*R* − *R*_min_)/(*R*_max_ − *R*). Where β*Kd = 722 nM, *R*_max_ = 3.94 and *R*_min_ = 0.28. See Hayat et al. (2003) for details.

### 2.7 Data analysis

M-current deactivation relaxations were fit with a biexponential function (*f*(*t*) = ∑*A*_*i*_ e^−*t*/τi^ + *C*) extrapolated to the beginning of the voltage step and the sum of the amplitudes of each component taken. Activation curves were individually fitted by the expression *y* = *A*1 – *A*2/(1 + e^(*x*-*x*0)*s*^) + *A*2 where *x*0 = half maximal conductance and *s* = slope. Parameters are given as mean ± SEM. Concentration inhibition curves were constructed by fitting the data from each cell to a Hill function (*y* = *V*_max_*(*x*^*n*^/IC_50_^*n*^ + *x*^*n*^)) and averaging the parameters log IC_50_, *n* (= slope) and *V*_max_ (usually set at 100%). GFP-PLCδ-PH measurements were obtained from a region of interest within the cytosol of the cell, avoiding the nucleus, and expressed as a percent of background subtracted basal fluorescence. Statistical tests used were two-tailed *t*-tests (paired or unpaired as appropriate) or ANOVAs with post hoc tests as stated.

### 2.8 Drugs and chemicals

Pertussis toxin, PMT, BAPTA-AM and Indo-1-AM were obtained from Calbiochem (Nottingham, UK). Wild type and the non mitogenic/non toxigenic C1165S mutant PMT was prepared as previously described (Ward et al., 1998). Cholera toxin, norepinephrine, oxotremorine M, angiotensin II, bradykinin, UTP and UDP were from Sigma and TTX from Tocris.

## 3 Results

### 3.1 PMT does not drastically alter Gq or G11 expression in SCG

**Fig. 1:**
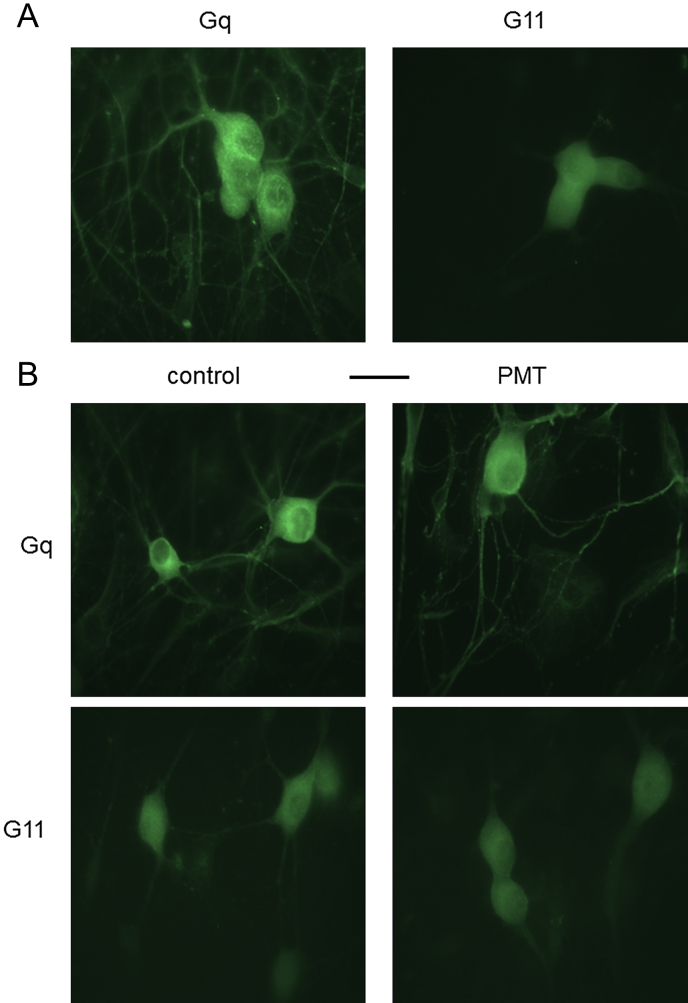
Immunostaining of rat SCG cells with antibodies to rat Gαq and Gα11. A: representative immunostaining of control cells for antibodies directed against Gαq or Gα11. These images were taken at the same camera exposure of 1.0 s to compare fluorescent staining of the two G-protein subtypes. B: comparative immunostaining for Gαq (upper row) and Gα11 (lower row) in control and PMT (500 ng ml^−1^, 18–24 h) pre-treated cells. Exposure times were 0.6 s and 2 s for Gαq and Gα11 respectively. The scale bar (25 μm) applies to all panels.

Using antibodies directed against rat Gαq or rat Gα11 we found both fluorescence for Gαq and Gα11 antibody labelling in control cells (Fig. 1A; *n* = four coverslips of each). This accords with previous mRNA measurements (Caulfield et al., 1994). When we compared the levels of fluorescence in control or PMT pre-treated cells we could see no striking differences in either the amount or distribution of the immunolabelling for either Gα11 or Gαq (Fig. 1 B).

### 3.2 PMT does not modify M-current amplitude or kinetics

Acute application (1–10 min) of PMT (100 ng ml^−1^) to sympathetic (SCG) neurons had no effect on the amplitude of the M-current (inhibition +1.2 ± 0.5%, *n* = 4). This is not surprising since the toxin acts intracellularly and takes a few hours to internalise (Lax et al., 2004). All of the following experiments were performed on cells that had been preincubated in the toxin (500 ng ml^−1^) for 18–24 h at 37 °C unless otherwise stated.

**Fig. 2:**
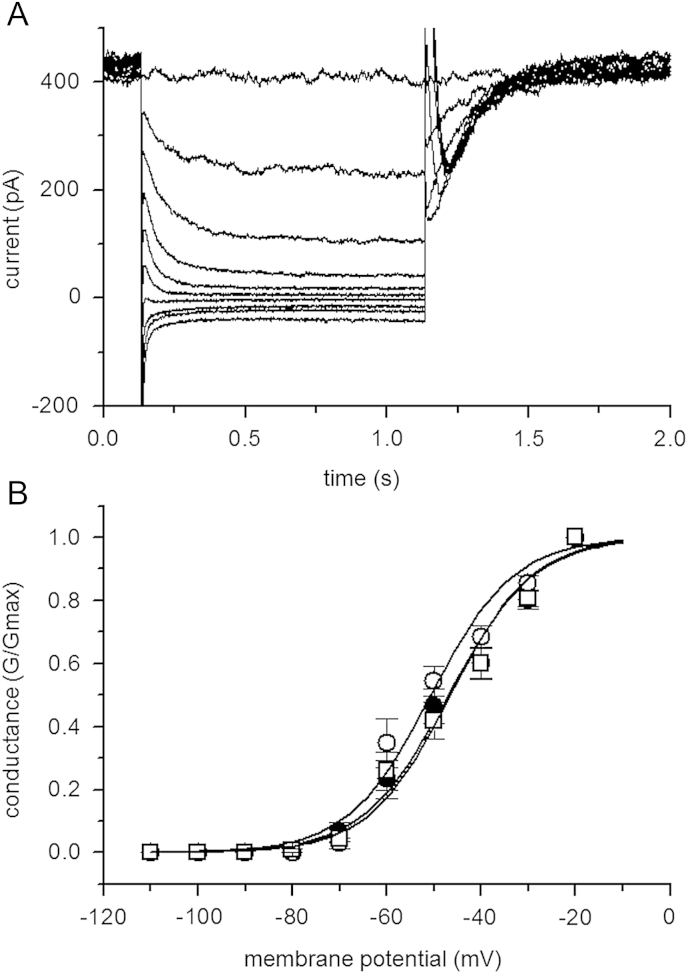
Preincubation with PMT or CTX has no effect on M-current activation curves. A) Example of a current–voltage relationship. Sympathetic SCG neurons were held at −20 mV to activate the Kv7/M-current then stepped negative every 30 s for 1 s in −10 mV increments to deactivate the current. Current traces show the time-dependent current decline as M-channels close. B) Activation curves measured from the current deactivations (see Methods) for control cells (open squares), cells preincubated in CTX (500 ng ml^−1^) for 18–24 h (open circles) or in PMT (500 ng ml^−1^) for 18–24 h (filled circles). Solid lines are Boltzmann fits to the mean data (see Methods and Materials); parameters of the curves are given in Results.

We compared the effect of preincubating SCG cells for 18–24 h in PMT (500 ng ml^−1^), or in cholera toxin (500 ng ml^−1^, as a negative control: Guo and Schofield, 2003), with control cells. The deactivation relaxation amplitudes of the M current (Fig. 2 A), measured at −50 mV, were not significantly different (ANOVA) between controls (201 ± 20 pA, *n* = 19), cells incubated in cholera toxin (202 ± 19 pA, *n* = 8) and cells preincubated in PMT (166 ± 16 pA, *n* = 18). Similarly, activation curves (Fig. 2B) were not different (one way ANOVA) after these treatments in terms of their half activation potentials (−46.6 ± 2.4 mV, −50.7 ± 2.1 mV and −47.0 ± 2.2 mV in 12 controls, 9 CTX treated and 11 PMT treated cells respectively) or slope values (8.70 ± 0.55, 8.83 ± 0.21 and 9.08 ± 0.58 mV e-fold^−1^). Maximum cellular conductances were 10.2 ± 1.3, 19.8 ± 2.9 and 12.8 ± 1.5 nS respectively. Thus, SCG neuron M-current is not directly affected by PMT or CTX.

### 3.3 PMT sensitises M-current to muscarinic acetylcholine receptor-mediated inhibition

**Fig. 3:**
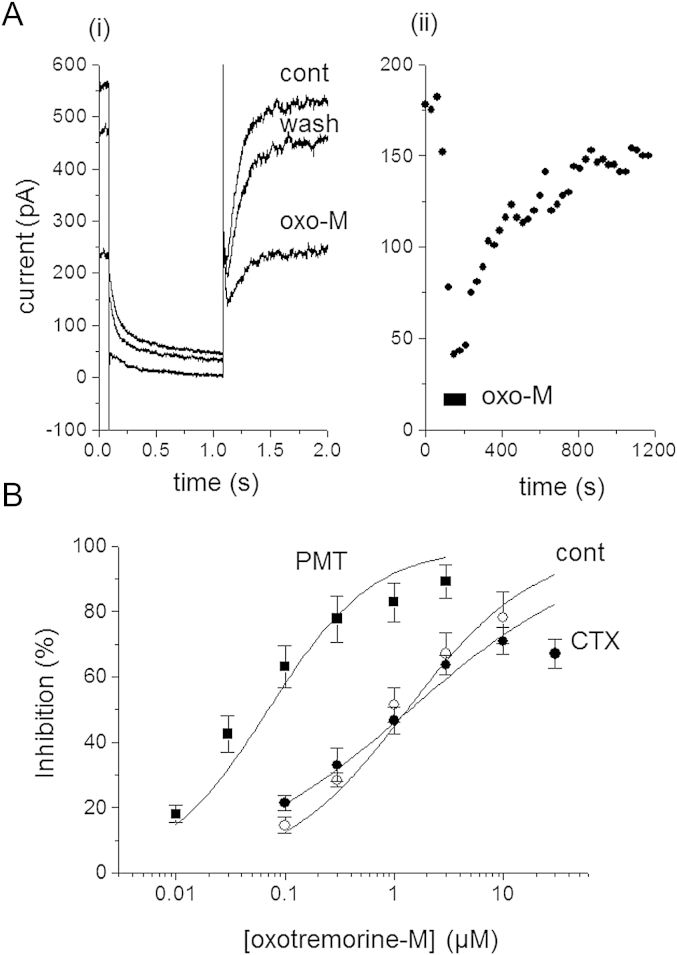
Preincubation with PMT increases the sensitivity of the M-current to inhibition by oxotremorine-M. A(i) example of a PMT-treated cell's response to oxotremorine-M. Records show current relaxations evoked by holding the cell at −20 mV and then stepping to −50 mV for 1 s. Trace cont: control; trace oxo-M: in the presence of 0.1 μM oxotremorine-M; trace wash: after washing in drug free solution. A(ii): time-course of the changes in deactivation current amplitudes illustrated in A(i). Measurements were made every 30 s. Oxotremorine-M application is shown by the solid bar beneath the points. B) Mean (SEM) concentration–inhibition curves in control cells (open circles), CTX treated cells (filled circles) and PMT treated cells (filled squares). Curves show least-squares Hill fits (solid lines) with the numerical data given in Table 1.

M-currents in rat SCG neurons are inhibited by muscarinic acetylcholine-receptor (mAChR) agonists such as oxotremorine-M (Oxo-M) acting on M1-mAChRs coupled mainly to Gq (Haley et al., 1998). Preincubation of cells in PMT (500 ng ml^−1^, Calbiochem) for 18–24 h shifted the Oxo-M concentration–inhibition curve 19-fold to the left compared with control and CTX-treated cells (Fig. 3 and Table 1). Very little, if any effect (1.7-fold shift) was seen with shorter incubation periods of 4–6 h (Table 1).

### 3.4 PMT does not alter angiotensin II or bradykinin-mediated inhibition of the M-current

**Fig. 4:**
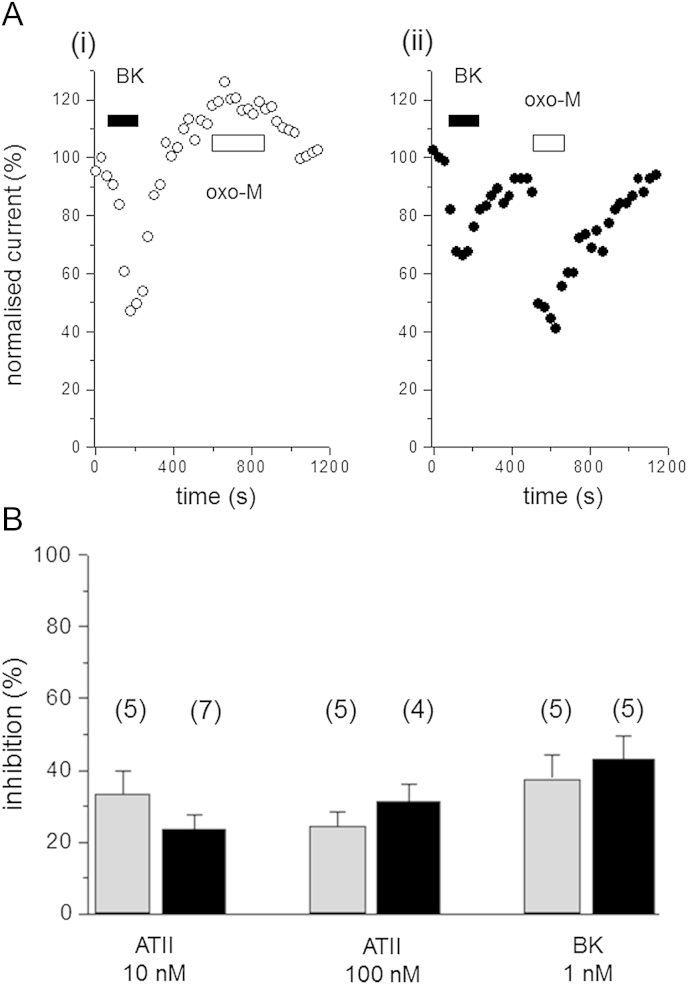
PMT alters M-current sensitivity to muscarinic receptor but not angiotensin or bradykinin receptor mediated inhibition. Comparison of the effect of 1 nM bradykinin (filled bars) and 100 nM oxotremorine-M (open bars) on M-current amplitude in control (A(i): open symbols) and PMT-pretreated (A(ii): filled symbols) SCG neurons. Note that the inhibition by oxotremorine-M is enhanced whereas inhibition by bradykinin was unaffected by PMT. M-currents were measured every 30 s as deactivation tail amplitudes using a voltage command to −50 mV from a holding potential of −20 mV for 1 s. B: % inhibition of M-current induced by angiotensin II (ATII, 10 and 100 nM) and bradykinin (BK, 1 nM) in control (light bars) and PMT pre-treated cells (dark bars). Numbers of cells in each group are indicated in brackets.

Both angiotensin (Constanti and Brown, 1981; Shapiro et al., 1994) and bradykinin (Jones et al., 1995) inhibit M-current in rat SGC neurons. In control cells, application of 1 nM bradykinin produced around 40% inhibition of the M-current and 100 nM oxotremorine had no effect (Fig. 4A). A cell pretreated with PMT (500 ng ml^−1^, 18–24 h) showed a similar response to bradykinin but an enhanced response to oxotremorine-M. Pooled data confirmed that the inhibition produced by 1 nM bradykinin in PMT treated cells (43.2 ± 6.3%) was not significantly different from that (37.6 ± 4.1%) in control cells (*n* = 5 for both). The inhibition produced by either 10 or 100 nM angiotensin II was also not altered significantly by PMT pre-treatment (Fig. 4B).

### 3.5 PMT sensitises GFP-PLCδ-PH translocation to muscarinic receptor activation

**Fig. 5:**
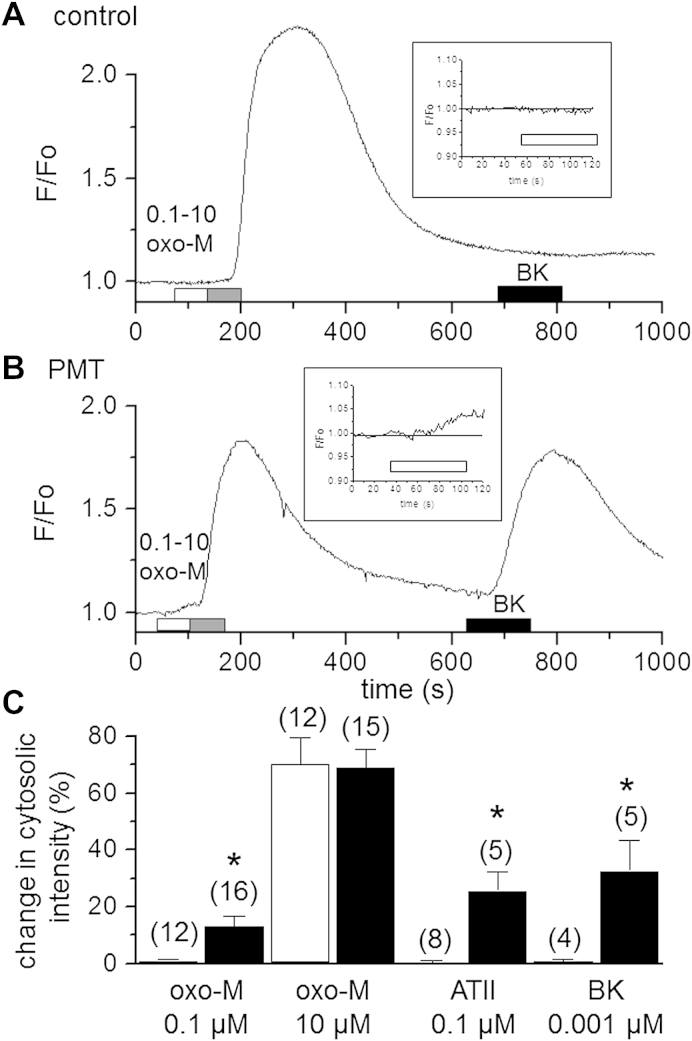
Effects of PMT on GFP-PLCδ-PH translocation. (A). Example of the membrane-to-cytosolic translocation of the GFP-PLCδ-PH construct (measured as increase in cytosolic fluorescence over basal fluorescence (F/F_0_) in a defined region of interest) in a control cell in response to 0.1 μM and 10 μM oxotremorine-M (open and light filled bar respectively) and to 1 nM bradykinin II (dark filled bar). Note: inset shows expanded trace of oxotremorine-M (0.1 μM) application. (B). Similar experiment to (A) but in a PMT (500 ng ml^−1^, 18–24 h) pre-treated SGC neuron. Drug applications and inset as in (A). (C). Pooled data showing % increase (mean ± SEM) over basal cytosolic fluorescence for two concentration of oxotremorine-M (0.1 and 10 μM), 0.1 μM angiotensin II and 1 nM (0.001 μM) bradykinin. Open bars: control cells; filled bars: PMT pre-treated cells. Numbers in brackets indicate number of cells tested and * indicates significant change (*P* < 0.05).

It has been suggested that an important mechanism for mAChR-mediated inhibition of M-current is the depletion of membrane phosphatidylinositol-4, 5-bisphosphate (PIP_2_) due to Gq-activated phosphoinositide turnover (Suh and Hille, 2002; Zhang et al., 2003; Suh et al., 2004; Winks et al., 2005; Robbins et al., 2006). Therefore we used the membrane-to-cytosol translocation of the fluorescent marker, GFP-PLCδ-PH as an index of phosphoinositide turnover (Stauffer et al., 1998; Varnai and Balla, 1998; Nahorski et al., 2003), to test if the enhanced sensitivity to muscarinic receptor stimulation could be detected upstream from channel closure. In control cells a sub-threshold concentration of oxotremorine-M (100 nM) did not produce any translocation of the probe (0.9 ± 0.7%, *n* = 12). However, in PMT treated cells there was a significant increase (*P* < 0.01) in translocation (12.9 ± 4.0%, *n* = 16; Fig. 5). At a maximal concentration of oxotremorine-M (10 μM) there was no difference in the translocation (control cells 70.1 ± 9.6%, *n* = 12; PMT-treated cells 68.9 ± 6.5%, *n* = 15). Similarly a submaximal (1 nM) concentration of bradykinin produced no significant translocation in control cells (0.6 ± 0.9%, *n* = 4) but a significant (32.5 ± 10.7%, *n* = 5; *P* < 0.05) translocation in PMT treated cells. Likewise, angiotensin (100 nM) caused no translocation in control cells (0.4 ± 0.8%, *n* = 4) but a significant (25.6 ± 6.8%, *n* = 5; *P* < 0.05) translocation in cells treated with PMT (see Fig. 5).

### 3.6 PMT does not alter Go transduction

**Fig. 6:**
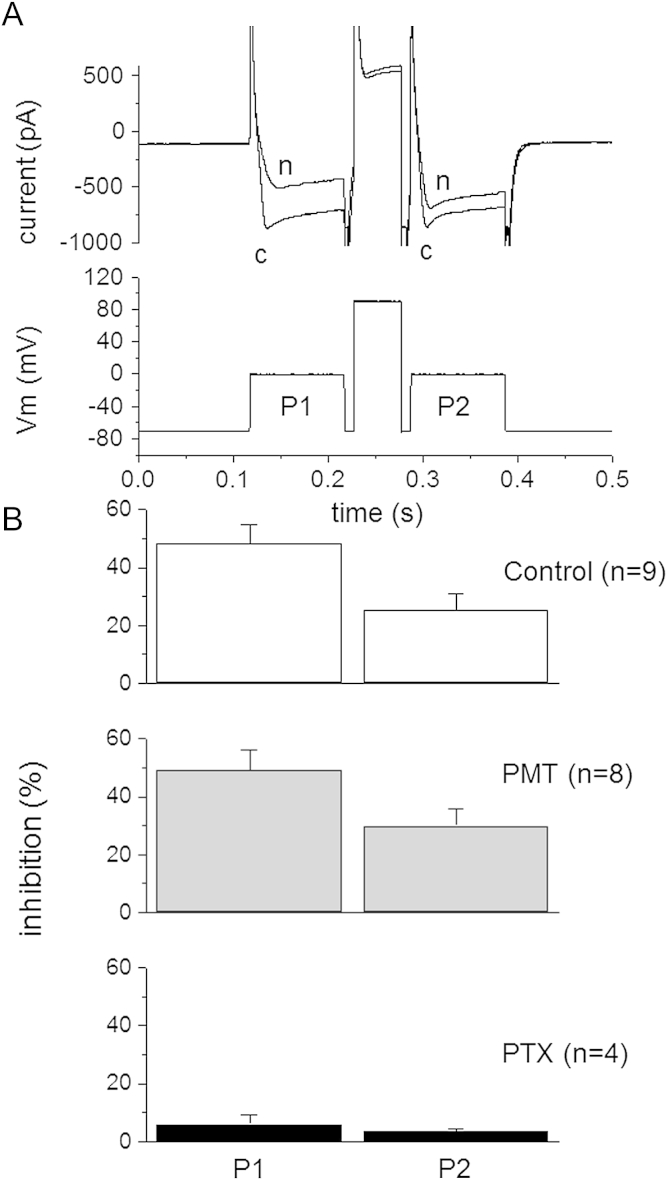
PMT does not alter Go mediated calcium channel current inhibition. (A). Example traces of calcium currents evoked by a twin-pulse protocol (c). Cells were voltage clamped at −70 mV and stepped to 0 mV for 100 ms before (P1) and after (P2) a 50 ms command pulse to +90 mV. In a control cell inhibition produced by norepinephrine (n, 10 μM) shows a strong block during P1 which is very much reduced after the +90 mV depolarising pulse at P2. The pooled data (B) shows that there was no difference in the inhibition between the control cells (filled bars) and those pretreated with PMT (500 ng ml^−1^ 18–14 h; shaded bars). On the other hand cells pretreated with *Pertussis* toxin (PTX, 500 ng ml^−1^, 18–24 h) showed almost complete abolition of the response, suggesting that the inhibition recorded under these conditions (whole cell and high calcium buffering) was mostly mediated by Go activation. The number of cells tested for each condition is given in brackets.

In order to test the action of PMT on a transduction pathway that does not involve Gi, Gq/G11 or G11/G12 we recorded calcium currents under conditions where the Go mediated, fast, voltage and pertussis toxin sensitive inhibition predominated. SCG neurons were patched in whole cell mode with 20 mM BAPTA added to the internal solution (Beech et al., 1991). Norepinephrine (10 μM) was used to inhibit the calcium current (Fig. 6A); under these conditions this inhibition (via α2-adrenoceptors) is solely mediated by Go (Caulfield et al., 1994; Haley et al., 1998; Delmas et al., 1998). Such inhibition is voltage-dependent, and is reduced by strong depolarisation (see Hille, 1994). Thus, as shown in Fig. 6B, inhibition was 48.3 ± 6.5% for the first pulse to 0 mV (P1) but was reduced to 25.2 ± 5.9% for the second pulse (P2) applied after an intervening step to +90 mV (*n* = 9). PMT pre-treatment (500 ng ml^−1^, 18–24 h) had no significant effect on either the degree of inhibition observed during the first pulse or the extent to which the inhibition was relieved by the depolarising step (P1 inhibition 49.2 ± 6.8%. P2 inhibition 29.9 ± 5.8%; *n* = 8). In contrast, in cells pretreated with *Pertussis* toxin PTX (500 ng ml^−1^, 18–24 h), inhibition was essentially abolished (P1 inhibition 6.0 ± 3.1%; P2 inhibition 3.5 ± 0.9%; *n* = 4).

### 3.7 Rat SCGs do not show calcium increases

Resting [Ca]_i_ in control SCGs was 77.7 ± 4.4 nM, (*n* = 46) and in PMT (500 ng ml^−1^, 18–24 h) treated cells it was not significantly different at 69.1 ± 6.4 nM (*n* = 19). Furthermore a significant calcium rise could not be detected following applications of oxotremorine-M (10 μM; see also (Cruzblanca et al., 1998; Delmas et al., 2002)), bradykinin (100 nM) or angiotensin II (100 nM) (0.6 ± 0.6 nM, (*n* = 5), −2.8 ± 6.1 nM (*n* = 11) and 1.3 ± 0.9 nM (*n* = 3) respectively). There was no change in the effect of these compounds after PMT pretreatment (500 ng ml^−1^, 18–24 h). Therefore we used NG108-15 cells in which we had already shown a robust calcium rise mediated by a range of receptors including M1 muscarinic (Robbins et al., 1993; Bowden et al., 1999) and P2Y_2_ purinergic (Chueh et al., 1993; Matsuoka et al., 1995; Czubayko and Reiser, 1996; Watano et al., 2002).

### 3.8 PMT amplifies M1 mAChR mediated calcium signals in NG108-15 cells

**Fig. 7:**
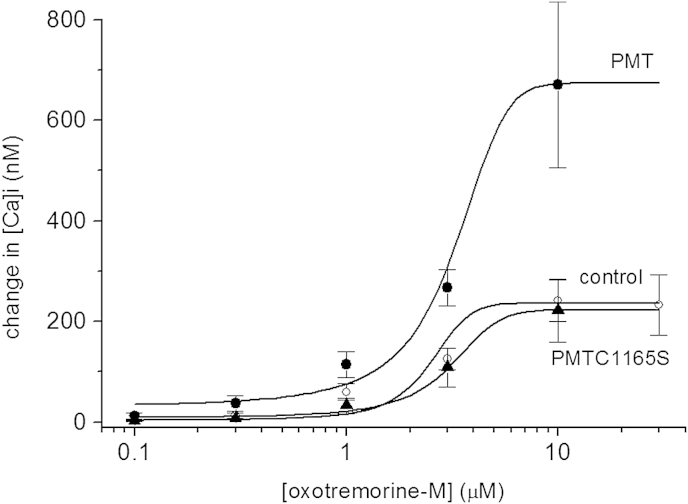
M1 muscarinic receptor mediated [Ca]_i_ increases in transformed NG108-15 cells are enhanced by PMT. Concentration response curves for oxotemorine-M evoked calcium increases in NG108-15 cells. Control cell (open circles, *N* = 10), PMT (500 ng ml^−1^, 18–24 h) pre-treated cells (filled circles, *N* = 6) and PMT C1165S (500 ng ml^−1^, 18–24 h) pre-treated cells (open triangles, *N* = 5). The points where fit with Hill curves as described in text.

As shown in Fig. 7 oxo-M concentration response curves were generated in control cells and cells incubated in PMT (500 ng ml^−1^) for 18–24 h. In control cells the oxo-M EC_50_ was 2.5 ± 0.3 μM with a maximum calcium rise of 236 ± 141 nM (*n* = 10). Cells exposed to PMT had a similar EC_50_ of 3.2 ± 0.3 μM but a significantly (*P* < 0.05, ANOVA) increased maximal response of 674 ± 167 nM (*n* = 6).

### 3.9 PMT increases resting [Ca]_i_ and amplifies calcium signals mediated by P2Y_2_ receptor activation in NG108-15 cells

NG108-15 cells express two purinergic receptors which can couple to phosphoinostide turnover, namely P2Y_6_ and P2Y_2_ (Sak et al., 2001). Under our conditions we found no responses to UDP (100 μM, *n* = 3) and consistent calcium signals to UTP (100 μM, *n* = 45) indicating that the response was mainly mediated by P2Y_2_ receptors that are insensitive to UDP. Furthermore the UTP calcium responses were not dependent on [Ca]_o_ or the action of voltage-gated calcium channels as calcium responses to UTP were 368 ± 71 nM in 2.5 mM [Ca]_o_, 363 ± 122 nM in 0 mM [Ca]_o_ and 365 ± 101 nM in CdCl_2_ (100 μM), *n* = 5 for each condition. These data suggest the response was predominantly mediated by calcium mobilisation from intracellular stores.

**Fig. 8:**
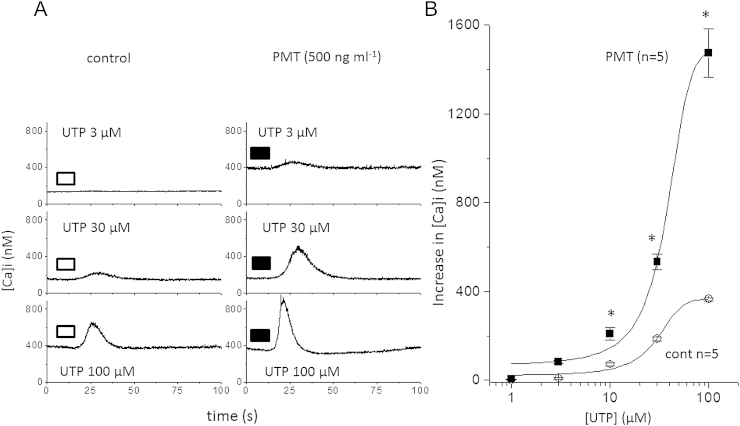
UTP concentration response curves showing responses in PMT treated cells compared to control cells. A) Example traces where bars indicate UTP application and B) concentration response curves of control (open circles) and 500 ng ml^−1^ (128-24 h) PMT treated (filled squares) NG108-15 cells. *N* = 5 for both groups of cells and * indicates *P* < 0.05 (*t*-test).

**Fig. 9:**
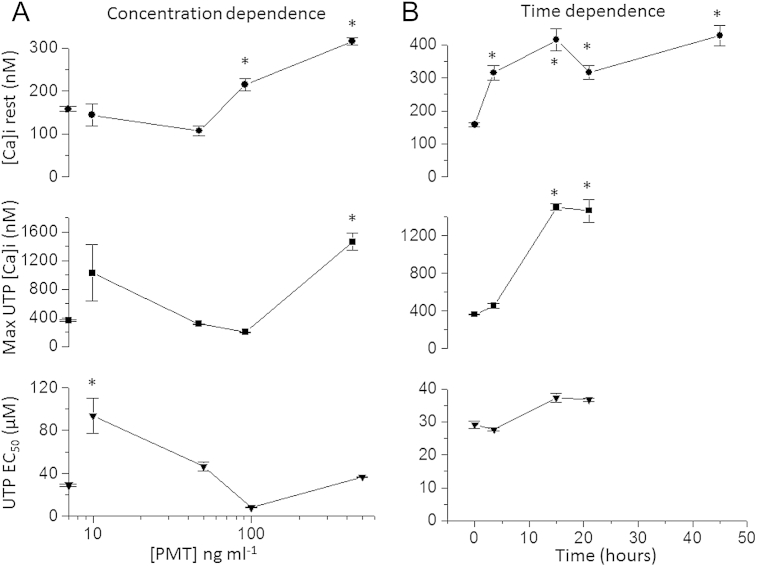
Concentration and time dependence of PMT on UTP induced calcium responses in NG108-15 cells. A) PMT concentration- and B) time-dependent changes in resting [Ca]_i_ (filled circles), maximal UTP evoked calcium response (filled squares) and UTP EC_50_ (filled triangles). The concentration dependence was done at 18–24 h and the symbols on the vertical axis of A indicate control values, in the absence of PMT. The time dependence was done at 500 ng ml^−1^. *N* = 5 for all points and * indicates *P* < 0.05 by ANOVA, compared to controls in A and time zero in B.

Similar to M1 mACh receptor calcium mobilisation in NG108-15 cells (Fig. 7), PMT (500 ng ml^−1^ for 18–24 h) enhanced P2Y_2_ calcium responses evoked by UTP (Fig. 8). This effect was studied in more detail in Fig. 9 where both the concentration- and time-dependence of PMT was measured on the resting [Ca]_i_, EC50 and maximal response of the UTP evoked calcium signals.

Of the three parameters measured the UTP EC_50_ was the most sensitive as a significant increase (*P* < 0.05, ANOVA, Dunnett post hoc test) was seen at 10 ng ml^−1^. Significant changes in resting [Ca]_i_ was next seen (100 ng ml^−1^) followed by maximal UTP response at the maximal concentration of 500 ng ml^−1^ (Fig. 9B). In terms of speed of response the resting [Ca]_i_ was the earliest event, within 3–4 h a significant increase was seen this was followed by an increase in UTP calcium response maximum at 12–24 h and a complete loss of response by 42–48 h (Fig. 9B).

### 3.10 Mutant PMT

To investigate whether it is the activation of Gαq or the phosphorylation of Gαq that is important for these effects we have used a mutant form of PMT, C1165S, which has lost its toxicity and mitogenicity but which does not appear to affect the endogenous phosphorylation of Gαq (Baldwin et al., 2003). SCG cells preincubated with 500 ng ml^−1^ PMT (C1165S) were compared with cells preincubated with wild-type toxin from the same source. As before, wild-type PMT (Lax) produced a 15-fold leftward shift in the oxotremorine-M concentration (M)Kv7 current inhibition curve, mean IC_50_ (95% confidence limits) 0.09 (0.05–0.16) μM without significantly altering the slope, mean (SEM): 1.25 ± 0.18, (*n* = 6). The C1165S mutant PMT (500 ng ml^−1^ for 18–24 h) produced no effect on the oxotremorine-M concentration (M)Kv-7 inhibition curve IC_50_ 0.30 (0.14–0.67) μM, slope 0.84 ± 0.16, (*n* = 6). Similarly the enhancement of the muscarinic receptor mediated calcium response in NG108-15 cells was not seen with the C1165S mutant PMT (Fig. 7). The cells exposed to PMT C1165S were not significantly different from control with the EC_50_ of 3.1 ± 0.2 μM and maximal response of 223 ± 62 nM (*n* = 5), suggesting that this mutant is inactive.

## 4 Discussion

### 4.1 Neuronal actions

To date studies on the nervous system or neuronal cells with PMT have been very limited. It has been demonstrated that PMT interacts with NG108-15 cell membranes by binding to phospholipids rather than gangliosides (Brothers et al., 2011). We have substantially added to this knowledge by showing that PMT can (whereas cholera toxin cannot) enhance the muscarinic receptor inhibition of Kv7.2 + 7.3 (M) current in rat SCG cells. However this effect is restricted to only some receptors that are known to regulate these channels. Interestingly the specificity of PMT is lost when we look at the upstream hydrolysis of PIP2 (Fig. 5). Here muscarinic ACh, angiotensin II and bradykinin receptor mediated responses are enhanced by PMT. This may highlight a difference in the regulation of Kv7.2 + 7.3 channels by these receptors (see later). PMT has no effect on the adrenoceptor mediated inhibition of voltage gated calcium currents, which are blocked by pertussis toxin (Fig. 6). In NG108-15 cells transfected with the gene for M1 muscarinic receptors the calcium mobilisation was enhanced as it was for endogenous purinergic P2Y_2_ receptors (Figs. 7 and 8). PMT is a potent toxin, with which we noted effects from 10 ng ml^−1^. The earliest effect of PMT was seen at 3–4 h, which was an increase in resting [Ca]_i_ followed by a dramatic increase in maximal response to UTP at 12–24 h, by 42–48 the response was abolished completely (Fig. 9). The time delay for the effect of PMT on calcium mobilisation is consistent with the lack of affect when applied acutely to SCG cells. The delay is likely to reflect the binding and internalisation of the toxin.

### 4.2 Direct effect on Kv7 (M) current

PMT also directly stimulates Gαq (Wilson et al., 1997; Baldwin et al., 2003; Orth et al., 2007), therefore we might have expected PMT itself to inhibit M-current. As show in Fig. 2, PMT had no direct effect on the Kv7 (M)-current in terms of amplitude or voltage dependence. This may suggest that its actions requires the activation of the G-protein, as often seen with compounds that block GTPase activity, such as GTP-γ-S (Robbins et al., 1993). In this case the PMT itself should have stimulated PIP2 hydrolysis, reduced membrane PIP2 and amplitude of the potassium current. The reason why this inhibition was not seen is likely to be due to the slow effect of PMT (Lax et al., 2004). The resultant PIP2 hydrolysis and rise in [Ca]_i_ (as seen in NG108-15 cells at 3–4 h incubation, Fig. 9A) can stimulate PI4 kinase (Koizumi et al., 2002) thereby sustaining membrane PIP2 levels (Winks et al., 2005; Zaika et al., 2006) which maintains the current amplitude. It is also possible that PMT applied in the absence of agonist(s) cannot activate Gq/G11 to an extent to deplete PIP2 enough to produce a significant inhibition of Kv(M)-current. It has been demonstrated that to get even the 18% (non-significant) inhibition in M-current observed in these experiments, PIP2 levels would have to fall by about 50% (Winks et al., 2005).

### 4.3 Gq and G11 molecular targets

Relatively recently the molecular targets of PMT have been identified (Orth et al., 2009, 2013; Babb et al., 2012). Deamination of homologous glutamine residues in G-protein alpha subunits of Gi, Gq, G12 and G13 reduces GTPase activity and leads to increased activation after receptor stimulation. For G11 it seems the situation is more complex. Originally it was thought that G11 was not a molecular target for PMT (Zywietz et al., 2001; Orth et al., 2004, 2007, 2009). More recently however this view has changed to suggest that G11 is also a target (Kamitani et al., 2011; Babb et al., 2012; Orth et al., 2013) albeit that PMT activation of G11 is weaker (Kamitani et al., 2011).

Both Gq and G11 are present in SCGs (Fig. 1) and NG108-15 cells (Williams and Kelly, 1993). The presence in SCGs of at least two of the five PMT sensitive G-proteins has been demonstrated here by immunocytochemistry and by others using functional antibodies (Caulfield et al., 1994; Jones et al., 1995) and antisense RNA (Haley et al., 1998). Consistent with mRNA measurements comparing Gq and G11 in SCG (Caulfield et al., 1994) and in cerebellar Purkinje cells (Hartmann et al., 2004) our immunostaining results show that Gq is more highly expressed than G11. However PMT caused no large alteration in the absolute or relative expression of either G-protein, indicating that the effect of PMT was not due to increased levels of G protein subunits. Our results are consistent with this in that the transduction pathways that were enhanced, Kv7.2 + 7.3 inhibition, in SCG neurones and calcium increased in NG108-15 cells, have been shown to be mediated by Gq and/or G11 G-proteins (Caulfield et al., 1994; Haley et al., 1998), whereas the pathway that do not involve a PMT sensitive G-protein (Go-mediated inhibition of calcium current; Delmas et al., 1998) was unaffected. There does however seem to be an inconsistency in that the Kv7(M)-current inhibition by angiotensin and bradykinin was not altered by PMT whereas the increased mobilisation of PIP2 was evident for all three receptors. For bradykinin, the explanation may be related to the fact that M-current inhibition produced by bradykinin (unlike that produced by oxotremorine-M) results not from PIP_2_ depletion but from the effects of Ca^2+^ ions released by the cytosolic product of PIP_2_ hydrolysis, IP_3_ (Cruzblanca et al., 1998; Gamper and Shapiro, 2003). Furthermore the bradykinin receptors and IP_3_ receptors responsible for this are organised into anatomically-discrete signalling ‘microdomains’, as opposed to the more diffuse location of the mAChRs (Delmas et al., 2002). However, not all of the bradykinin receptors are confined to these microdomains (Delmas et al., 2002), and those in the rest of the cell membrane would be expected to couple avidly to the abundant Gq protein, just like the diffusely-distributed mAChRs. Their effect may dominate the globally-observed PIP_2_ hydrolysis as visualised with the GFP-PLCδ-PH construct, and hence become enhanced by PMT, but this would not contribute to M-current inhibition since it may not be associated with significant PIP_2_ depletion (see Gamper et al., 2005; Winks et al., 2005) and would not generate a Ca^2+^ signal (Delmas et al., 2002). As further evidence for a dissociation between global PIP_2_ hydrolysis and M-current inhibition by bradykinin, the concentration of bradykinin required to induce translocation of GFP-PLCδ-PH is substantially above that which inhibits M-current: the IC_50_ for the latter is about 1 nM (Jones et al., 1995; see also Fig. 4), whereas concentrations above this are needed to translocate GFP-PLCδ-PH (Winks et al., 2005, and Fig. 5). In contrast, the concentration–response curves for the two readouts of mAChR-effects are virtually identical (Winks et al., 2005). In the recent work elegantly quantifying and modelling both arms of the Gq signalling pathway (Dickson et al., 2013; Falkenburger et al., 2013) it is seen that calcium increases are saturated by the IP3 generated from small amounts of PIP2 hydrolysis. This then means that the PMT mediated increase in PIP2 hydrolysis monitor by GFP-PLCδ-PH would not substantially increase PIP2 depletion and thereby not enhance M-current inhibition produced by bradykinin.

We rather assume that the mechanism for M-current inhibition by angiotensin II is more similar to that for bradykinin rather than for oxotremorine-M, as judged from the comparable effects of PMT on the two peptides. Although there is evidence that angiotensin II may behave more like muscarine than bradykinin (Zhang et al., 2011), the identity of its cognate G-protein has not been determined.

### 4.4 Mutant PMT

We have demonstrated in two cell types that the PMT C1165S mutant does not significantly shift the concentration inhibition curves for muscarinic receptor inhibition of Kv7 (M)-current in SCG nor does it enhance the muscarinic receptor evoked calcium rise in NG108-15 cells (Fig. 7). This may suggest that C1165 plays an important part in the deamidation of the G-proteins and that tyrosine phosphorylation is not a necessary step in the activity of PMT (Ward et al., 1998).

### 4.5 Other G-proteins

We have demonstrated under conditions that favour Go mediated inhibition of voltage-gated calcium current that PMT is ineffective. Although it is possible that a proportion of this inhibition could be mediated by Gi beta/gamma subunits (Delmas et al., 1999) however the proportion of voltage insensitive inhibition did not alter significantly in the presence of PMT indicating that either there was minimal Gi mediated inhibition or less likely that PMT does not activate Gi under these conditions. At present the effects we have shown can be explained predominantly by activation of Gq and/or G11. However there are other PMT sensitive G-proteins in the cells we have used in this study. For example NG108-15 cells express G12 and G13 (Spicher et al., 1994) as well as Gi2 and Gi3 (Wilk-Blaqszczak et al., 1996) so it would be expected that other signalling pathways are also being activated.

### 4.6 Conclusions

Kv7.2 + 7.3 channels are located in critical parts of neurones in both peripheral and central nervous systems, namely the nodes of Ranvier and the axon initial segment (Devaux et al., 2004; Schwarz et al., 2006; Hu et al., 2007). Functional deficits of these channels mediated by genetic mutations (BFNS) or blockade by drugs directly or toxins indirectly can lead to hyperexcitability and ultimately seizures. Increases in intracellular calcium can have multiple effects including increasing neuronal excitability. Therefore, although not all human isolates of *P*. *multocida* are toxigenic, we would predict that PMT toxicity in the CNS of humans would present with hyperexcitability symptoms such as seizures. For PMT to have a direct effect on the brain, it must cross the blood brain barrier. At present there is no direct evidence for or against this in animals or humans. Indeed seizures reported in meningitis may be mediated by a mechanism other than a toxigenic effect on the neurones.

## Conflicts of interest

The authors declare no conflicts of interest.

## Figures and Tables

**Table 1:** Effects of PMT on the oxotremorine-M concentration-M(Kv7) current inhibition curves.

Toxin	Source	Preincubation time (hours)	IC_50_ (μM)	Slope	*N*
Control	–	–	1.32 (0.65–2.67)	0.75 ± 0.16	6
CTX	Sigma	18–24	1.39 (0.55–3.54)	0.50 ± 0.05	7
PMT	Calbiochem	18–24	0.07 (0.02–0.22)	0.90 ± 0.15	9
PMT	Calbiochem	4–6	0.76 (0.13–4.29)	0.75 ± 0.16	6
PMT	Lax	18–24	0.09 (0.05–0.16)	1.25 ± 0.18	6

The data from each cell was fitted to a Hill equation (see Materials and Methods). The IC_50_s are given as the geometric mean with 95% confidence limits in brackets and slope values are given as the mean ± SEM. Maximal inhibition was constrained to 100% and *N* is the number of cells tested.
